# Conservatively Managed Chronic Bronchopleural Fistula After Lung Cancer Tri-Modality Therapy: A Case Report

**DOI:** 10.1016/j.adro.2021.100811

**Published:** 2021-09-29

**Authors:** Nilanjan Haldar, Christian Fernandez, Nathaniel R. Evans, Maria Werner-Wasik

**Affiliations:** a Departments of Radiation Oncology; b Cardiothoracic Surgery, Thomas Jefferson University, Philadelphia, Pennsylvania

## Introduction

The standard of care for locally advanced non-small cell lung cancer is definitive chemoradiotherapy followed by adjuvant immunotherapy.^1^ The addition of surgery to the treatment paradigm is thought to provide further benefit in carefully selected patients, when surgery is limited to lobectomy.^2^^,^^3^ Bronchopleural fistula (BPF) is a known but rare complication of lung cancer resection, with an incidence of <3% reported in the literature. Neoadjuvant radiation therapy is thought to be a risk factor.^4^ BPF most commonly occurs within the first several weeks after surgery but can occur anytime postoperatively and is generally managed with surgical correction.^5^ Herein, we present a case of a 48-year-old woman treated with tri-modality therapy who developed a BPF 2 years after initial therapy and was managed conservatively.

## Case Presentation

**Figure 1:**
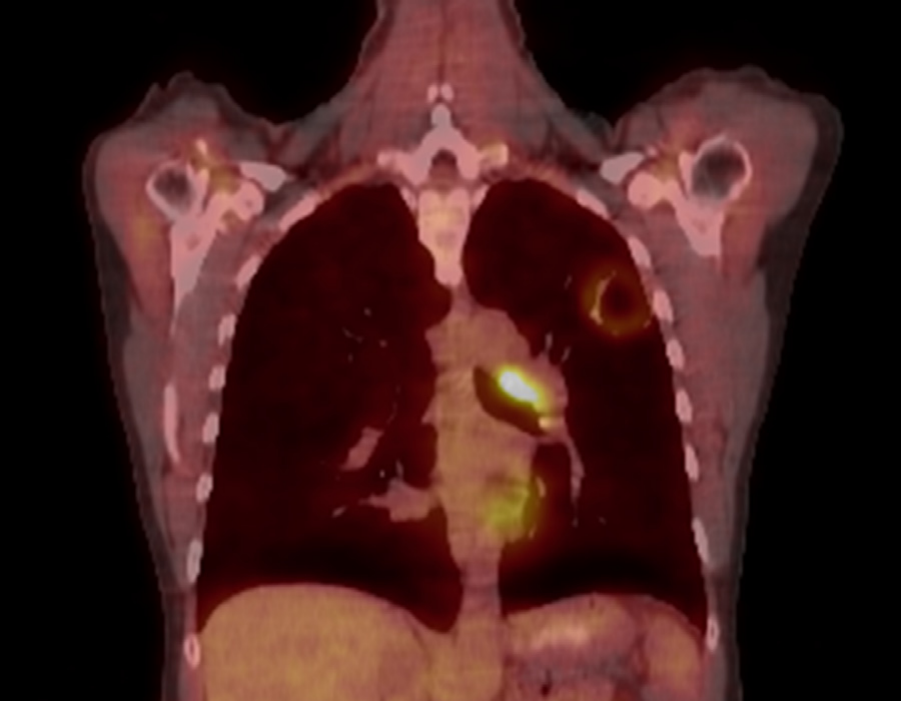
Presenting positron emission tomography**/**computed tomography. Representative positron emission tomography/computed tomography coronal slice of the left upper lobe cavitary adenocarcinoma with hyper-metabolic rind and nonbulky lymphadenopathy.

A 48-year-old woman active smoker with an 80 pack-year smoking history presented with shortness of breath, cough, low-grade fevers, night sweats, and weight loss. On physical examination, she was found to be febrile with digital clubbing and no findings on auscultation. Computed tomographic (CT) scan of the chest revealed a 3.3-cm cavitary lesion of the left upper lobe. Initial fine-needle biopsy was inconclusive; however, 3 months later endobronchial ultrasound-guided biopsy was performed and the patient received a diagnosis of poorly differentiated adenocarcinoma. Positron emission tomography/CT revealed an avid cavitary primary and nonbulky lymphadenopathy (Fig. 1). Magnetic resonance imaging of the brain was negative (stage IIA cT2N2M0). Her case was presented at multidisciplinary tumor board and treatment with neoadjuvant chemoradiotherapy followed by lobectomy was recommended.

## Treatment

**Figure 2:**
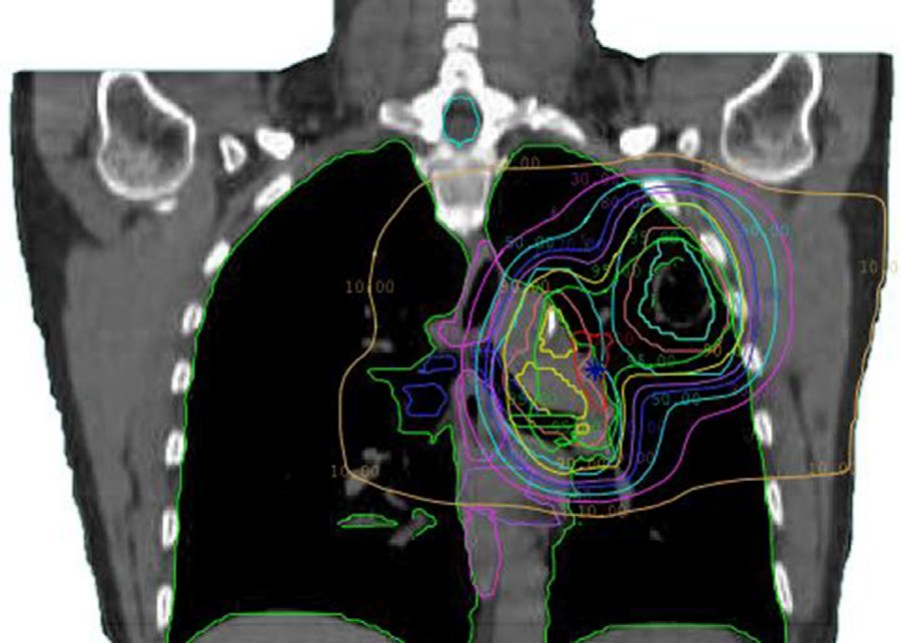
Intensity modulated radiation therapy treatment plan. Radiation plan of the left upper lobe and mediastinal adenocarcinoma with representative coronal plane. Prescription dose was 60 Gy in 2 Gy fractions to the 90% isodose line.

She received 60 Gy delivered in 30 fractions to her primary and mediastinal nodal disease with intensity modulated radiation therapy prescribed to the 90% isodose line with concurrent cisplatin and etoposide. The planning treatment volume received a maximum dose of 66.7 Gy and a mean dose of 63.8 Gy. The volume of uninvolved lung receiving greater than or equal to 20 Gy (V20Gy) was 22%. The ipsilateral left main stem and branches of the bronchus were within the planning treatment volume and received full dose (Fig. 2). She tolerated therapy well with only grade one dermatitis, fatigue, and pneumonitis. Two months after chemoradiotherapy, the patient underwent thoracotomy, left upper lobectomy, mediastinal lymph node dissection, and intercostal muscle flap buttress to the bronchial stump. Postsurgical pathology revealed pathologic complete response to neoadjuvant therapy, with no tumor detected in the left upper lobe or dissected lymph nodes. She recovered from surgery without complications and was discharged after 3 days. Throughout the next 2 years, CT surveillance scans were performed and notable for apical postradiation fibrosis. The patient continued to smoke.

## Bronchopleural Fistula

**Figure 3:**
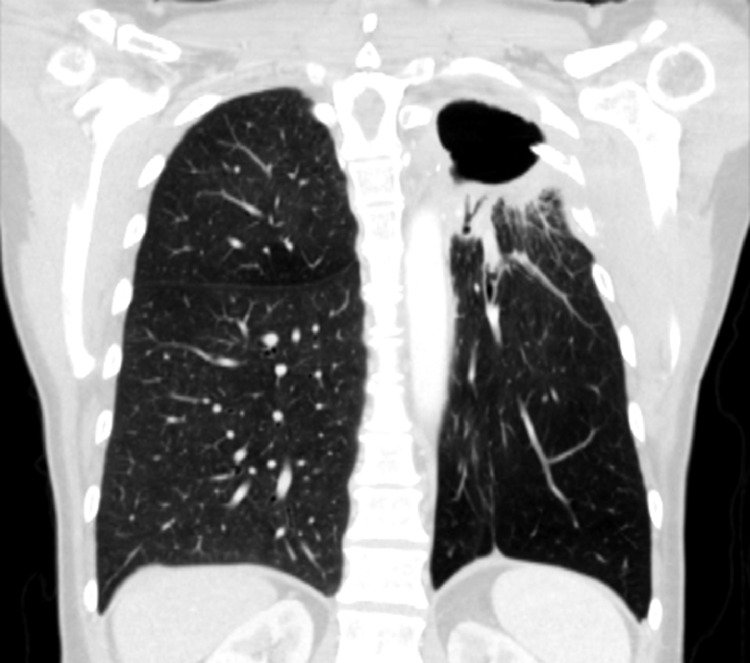
Bronchopleural fistula. Representative coronal computed tomography slice of the peripheral bronchopleural fistula 2 years after tri-modality therapy.

The patient presented to sports medicine due to acute on chronic left shoulder pain. A left shoulder magnetic resonance imaging was ordered, revealing a left pneumothorax, nondisplaced left-sided rib fractures, as well as tendinosis of the rotator cuff. The patient denied a history of fall or other trauma. Due to concern for recurrent malignancy, a positron emission tomography/CT was performed which demonstrated a hypermetabolic rind around the surgical cavity with extension into the pleural space. An ultrasound-guided biopsy was negative for malignancy, showing chronic inflammatory changes and fibrovascular tissue. Subsequent CT-guided biopsy confirmed these findings. Her case was reviewed at multidisciplinary tumor board, and the findings were determined to be due to postradiation therapy changes. She continued CT surveillance for the next 6 months with interval development of an asymptomatic, loculated hydropneumothorax in the left lung apex, and peripheral BPF 24 months after surgery (Fig. 3). One-way valve placement was considered, however, given that she was asymptomatic, continued surveillance imaging was elected. Subsequent CT imaging for the next year demonstrated stable to slightly improved hydropneumothorax. She continues to be under surveillance with no evidence of disease 4 years from treatment, nor intervention 24 months from BPF diagnosis. She continues to smoke, although has made significant progress toward cessation.

## Discussion

BPFs are defined as a communication between the lobar, segmental, or major bronchus and the pleural space and are most commonly associated with surgical procedures.^6^ They can be either central or peripheral, with central BPFs being defined as communications between the pleura and larger branches of the tracheobronchial tree, and peripheral BPFs as communications with the pulmonary tree distal to the segmental bronchi.^7^ It follows then that central BPFs are more often a complication of pulmonary surgery and can be detected on bronchoscopy. Peripheral BPFs can have etiologies in addition to surgery such as abscess, bronchiectasis, pneumonia, tuberculosis, neoplasms and radiation, and largely depend on imaging for detection, with small peripheral BPFs being potentially more difficult to diagnose.^8^ The most common cause for BPFs is surgical lung resection with lobectomies reporting a lower incidence rate (0.5%-1.2%) compared with pneumonectomies (4.5%).^9^^,^^10^ Central BPFs can lead to life-threatening pneumothorax and infection and can be a source for significant morbidity and mortality.^5^

Chemoradiotherapy is thought to increase the risk of postsurgical BPF. Li et al identified neoadjuvant radiation (odds ratio [OR] 3.914; *P* = .009) and chemoradiotherapy (OR 2.166; *P* = .004) as significant independent risk factors for developing BPF, and neoadjuvant chemotherapy alone was not found to be significantly associated with BPF (OR 1.857; *P* = .104).^4^ The onset of BPF is defined as early if occurring within 1 to 7 days after surgery, intermediate from 8 to 30 days, and late after 30 days. The incidence of late-onset BPF is rare and varies from months to years after surgery.^11^^,^^12^ Although the pathophysiology is still unclear, decreased bronchial mucosal blood supply, impaired healing, and inflammation after chemoradiotherapy are thought to play a role.^13^ In addition, our patient's continued active smoking status suggests a concurrent active inflammatory process that may have attributed to her late-onset BPF, as chronic inflammation has been shown to increase the risk of BPF, reinforcing the importance of smoking cessation even after diagnosis and treatment.^14^^,^^15^ Given the difficulty of radiographically diagnosing small peripheral BPFs, and the need to contextualize findings relative to the surgical and radiation therapy fields, multidisciplinary evaluation including all members of the treatment team is critical to not over or underdiagnose BPFs after lung cancer surgery.

Currently, no clinical consensus or guidelines for the treatment of BPF exist; however, management usually begins with the treatment of life-threatening complications of BPF including antibiotics, chest tube placement, and ventilation, followed by fistula occluding procedures or surgical correction.^9^^,^^12^^,^^14^, ^15^, ^16^ Conservative management can be a viable strategy for a subset of postlobectomy patients with minor peripheral BPFs, occasionally resulting in spontaneous fistula closure, although it is ambiguous if this strategy is effective for peripheral BPF detected on CT.^8^^,^^16^ Our case was of a rare late peripheral BPF found incidentally on imaging in an asymptomatic patient. To the best of our knowledge, current recommendations are limited to acute or symptomatic presentations, with no published cases of late peripheral BPF after tri-modality therapy managed with surveillance.^12^^,^^16^ Due to the asymptomatic and uncomplicated nature of her presentation, contrasted with the potential of complications from an additional procedure or surgical intervention, surveillance was elected. Our case emphasizes the need for multidisciplinary evaluation of lung cancer patients to cater treatments and interventions to their unique circumstances.

## Conclusion

We present the case of a locally advanced lung cancer patient successfully treated with tri-modality therapy with a rare, delayed complication of BPF managed conservatively. Patients undergoing neoadjuvant radiation therapy for lung cancer, particularly those that continue to smoke, are at increased risk of developing BPF even years after treatment. Treatments for late BPF in patients with lung cancer should be tailored to the acuity of symptoms and unique patient factors, and are best determined in a multidisciplinary manner.
